# Using inflammatory indexes and clinical parameters to predict radiation esophagitis in patients with small-cell lung cancer undergoing chemoradiotherapy

**DOI:** 10.3389/fonc.2022.898653

**Published:** 2022-11-22

**Authors:** Jianjian Qiu, Dongmei Ke, Hancui Lin, Yilin Yu, Qunhao Zheng, Hui Li, Hongying Zheng, Lingyun Liu, Jiancheng Li

**Affiliations:** Clinical Oncology School of Fujian Medical University, Fujian Cancer Hospital, Fuzhou, China

**Keywords:** small cell lung cancer, radiation esophagitis, inflammation index, nomogram model, radiation therapy

## Abstract

**Objective:**

Radiation esophagitis (RE) is a common adverse effect in small cell lung cancer (SCLC) patients undergoing thoracic radiotherapy. We aim to develop a novel nomogram to predict the acute severe RE (grade≥2) receiving chemoradiation in SCLC patients.

**Materials and methods:**

the risk factors were analyzed by logistic regression, and a nomogram was constructed based on multivariate analysis results. The clinical value of the model was evaluated using the area under the receiver operating curve (ROC) curve (AUC), calibration curves, and decision curve analysis (DCA). The correlations of inflammation indexes were assessed using Spearman correlation analysis.

**Results:**

Eighty-four of 187 patients (44.9%) developed grade ≥2 RE. Univariate analysis indicated that concurrent chemoradiotherapy (CCRT, p < 0.001), chemotherapy cycle (p = 0.097), system inflammation response index (SIRI, p = 0.048), prognostic-nutrition index (PNI, p = 0.073), platelets-lymphocyte radio (PLR, p = 0.026), platelets-albumin ratio (PAR, p = 0.029) were potential predictors of RE. In multivariate analysis, CCRT [p < 0.001; OR, 3.380; 95% CI, 1.767-6.465], SIRI (p = 0.047; OR, 0.436; 95% CI, 0.192-0.989), and PAR (p = 0.036; OR, 2.907; 95% CI, 1.071-7.891) were independent predictors of grade ≥2 RE. The AUC of nomogram was 0.702 (95% CI, 0.626-0.778), which was greater than each independent predictor (CCRT: 0.645; SIRI: 0.558; PAR: 0.559). Calibration curves showed high coherence between the predicted and actual observation RE, and DCA displayed satisfactory clinical utility.

**Conclusion:**

In this study, CCRT, SIRI, and PAR were independent predictors for RE (grade ≥2) in patients with SCLC receiving chemoradiotherapy. We developed and validated a predictive model through these factors. The developed nomogram with superior prediction ability can be used as a quantitative model to predict RE.

## Introduction

Lung cancer is one of the most common malignant tumors and the leading cause of cancer death globally (1). Small cell lung cancer (SCLC) accounts for 15% of all lung cancers characterized by rapid doubling time, early metastasis, and poor prognosis (2). According to the Veterans Administration Lung Cancer Study Group Classification, SCLC can be divided into limited-stage small cell lung cancer (LS-SCLC) and extensive-stage small cell lung cancer (ES-SCLC). Because small cell lung cancer is difficult to diagnose early, only 2-5% of patients can be treated by surgery. And SCLC is sensitive to chemotherapy and radiotherapy, so the primary treatment for most patients is chemoradiotherapy (3). Nevertheless, radiation esophagitis (RE) is a common adverse reaction in SCLC patients who receive radiation therapy. In radiotherapy for lung cancer, it is impossible to avoid esophageal irradiation completely because of several factors: large, irregular shape and central location of lung cancer, often involving mediastinal lymph nodes (LN), and the central location and length of the esophagus (4, 5). Despite advances in radiotherapy technology, RE is still a common side effect in patients receiving chemoradiotherapy.

RE usually occurs within two months from the beginning of radiotherapy to the end of radiotherapy. The typical symptoms of RE are dysphagia, retrosternal pain, burning, and other symptoms. Some patients may also have esophageal perforation or esophageal tracheal fistula and other serious complications. The development of RE will affect the quality of life of patients and the efficacy of local tumor control and treatment. Therefore, it may lead to hospitalization or interruption, or early termination of treatment, and the inability to eat requires a parenteral feeding tube in severe cases (6). In a randomized trial, 21% of patients receiving concurrent chemoradiotherapy (CCRT) stopped treatment because of severe RE (7). Predicting RE allows clinicians to take appropriate preventive measures in advance, such as medication, dietary guidance, rehydration, or tube feeding. Identifying low-risk patients with RE provides an opportunity to increase the dose of radiotherapy to improve tumor control. Hence, identifying predictive factors before the onset of RE may contribute to early intervention to decrease or avert the occurrence of severe RE (8).

Although some predictors of RE have been identified, including dosimetric factors and patient characteristics (9–11), it remains unclear how these factors can be used in routine clinical practice. Moreover, predictive models should at least perform better than doctors themselves to aid treatment decisions. However, this has been poorly studied in small cell lung cancer to the best of our knowledge. Consequently, we decided to build a nomogram model using clinical, dosimetric factors, and inflammation index to predict RE in SCLC patients receiving chemoradiotherapy.

## Materials and methods

### Patients

Between December 2008 and June 2020, 187 SCLC patients who received chemoradiotherapy at the Fujian Provincial Cancer Hospital were retrospectively reviewed. Inclusion criteria for eligible patients were: (A) Pathologically confirmed SCLC; (B) Patients receiving chemoradiotherapy; (C) There is a complete medical record of RE; (D) Received conventionally fractionated radiotherapy with platinum-based chemotherapy; (E) Availability of clinical and inflammatory data. Exclusion criteria were: (A) Patients had previously received chest radiotherapy for some reason; (B) Underwent pneumonectomy; (C) Failed to participate in the whole course of radiotherapy. (D) Hyper-fractionated radiotherapy was administered. Finally, a total of 187 patients were enrolled. The Ethics Committee approved this study at Fujian Provincial Cancer Hospital (K2021-115-01).

### Treatment schedules

All patients were scheduled to receive intensity-modulated radiation therapy (IMRT) or 3-dimensional conformal radiation therapy (3D-CRT). The prescribed RT dose was 46 - 70 Gy, 23 - 35 fractions (once-daily), 5 days per week. Patients were positioned by computed tomography (CT) simulation with a postural fixation device. Contrast-enhanced CT scans cover the entire chest from the cricoid cartilage to the costal diaphragmatic angle (the range can be increased according to the tumor) with a thickness of ≤ 5 mm. Radiotherapy (RT) was performed using a 6MV medical linear accelerator. According to the ESTRO ACROP guidelines, the gross tumor volume (GTV) includes lung primary tumors and involved or elective lymph nodes. The clinical tumor volume (CTV) is obtained by expanding 5 mm in all directions based on GTV. The planned tumor volume (PTV) is obtained by expanding 5-8 mm in all directions based on CTV (12). Dose and volume limits for organs at risk (OARs) were based on Radiotherapy and Oncology Group (RTOG) guidelines.

The 187 eligible patients received individualized concurrent or sequential chemoradiotherapy. The chemotherapy regimens included etoposide, irinotecan, or paclitaxel with cisplatin, carboplatin, lobaplatin, or nedaplatin. Most patients had received etoposide 100 or 120 mg/m2 day1-3 with cisplatin 75 or 60 mg/m2 day1 or carboplatin 80 or 60 mg/m2 day1 chemotherapy regimens. Every three weeks was a cycle. The chemotherapy regimens followed the National Comprehensive Cancer Network (NCCN).

### Blood and biochemical parameters

Inflammation and nutritional index were calculated based on blood and biochemical parameters. For example, systemic immune-inflammation index (SII): absolute neutrophil count times absolute platelet count divided by absolute lymphocyte count. system inflammation response index (SIRI): absolute neutrophil count times absolute monocyte count divided by absolute lymphocyte count. neutrophil-lymphocyte ratio (NLR): absolute neutrophil count divided by absolute lymphocyte count. platelets-lymphocyte radio (PLR): absolute platelet count divided by absolute lymphocyte count. prognostic-nutrition index (PNI): serum albumin level plus five times absolute lymphocyte count. platelets-albumin ratio (PAR): absolute platelet count divided by serum albumin level. The blood biochemical data were collected five days before therapy.

### Evaluation of radiation esophagitis

Toxic side effects were assessed weekly for each patient during RT and monthly follow-up for three months after completion of radiotherapy. Radiation esophagitis (RE) was diagnosed by radiation oncologists based on clinical symptoms and imaging evidence. RE was assessed and graded according to the Radiation Therapy Oncology Group scale (RTOG)/European Organization for Research and Treatment of Cancer (EORTC). The highest grade of esophagitis was recorded during treatment and follow-up. In this study, only grade ≥ 2 radiation esophagitis was considered an endpoint event.

### Statistical analysis

In this study, all continuous variables are converted into classification variables according to the optimal cut-off value of the Receiver Operating Curve (ROC). The risk factors for grade 2 or higher radiation esophagitis (RE) were identified by univariate logic regression analysis. Risk factors with p < 0.10 in univariate analysis were incorporated into the multivariate logistic regression analysis to determine independent predictors of the occurrence of RE. The area under the ROC curve (AUC), calibration curve (with 1000 bootstrap resamples), and decision curve analysis (DCA) were used to assess the clinical utility of the nomogram. The AUC value of the nomogram was greater than that of each independent predictor, indicating a preferable discrimination ability. The calibration curve was used to assess the agreement between the actual and predicted probability of occurrence of RE. DCA was used to evaluate the clinical benefit of the nomogram by quantifying net benefits at different threshold probabilities. The correlations of inflammation indexes were assessed using the Spearman correlation analysis. All statistical analyses were performed using SPSS software (version 25.0) and R software (version 4.0.2). All p values were double-tailed, and p < 0.05 was considered statistically significant.

## Results

### Patient characteristics and incidence of RE

Baseline characteristics of 187 eligible patients are presented in **Table 1**. One hundred and seventy-four patients (93.0%) were male, and thirteen patients (7.0%) were female. The median age of patients was 60 years. A total of 94 patients were smokers, and 93 patients were non-smokers. Of all patients receiving chemoradiotherapy, seventy-seven patients (41.2%) received concurrent chemoradiotherapy. The median chemotherapy cycle of a platinum-based chemotherapy regimen was four cycles. Most of the patients (68.4%) were limited-stage small cell lung cancer. The median duration of radiotherapy was 39 days, and the median RT dose was 57.2Gy. The optimal cut-off values of SII, SIRI, NLR, PNI, PLR, and PAR were 415.2, 0.3, 1.85, 49.5, 231.1, and 8.0, respectively. In general, grade ≥ 2 RE incidence was 44.9% (84/187) in patients with SCLC undergoing chemoradiotherapy.

**Table 1:** Baseline characteristics of patients.

Variable	Total (N)	Percentage (%)
Gender		
Male	174	93.0%
Female	13	7.0%
Age (years)		
<60	85	45.5%
≥60	102	54.5%
Smokers		
No	93	49.7%
Yes	94	50.3%
RE		
grade ≤1	103	55.1%
grade≥2	84	44.9%
CCRT		
No	110	58.8%
Yes	77	41.2%
Chemotherapy cycle		
<4	81	43.3%
≥4	106	56.7%
Stage		
Limited	128	68.4%
Extensive	59	31.6%
Duration of radiotherapy (day)		
<39	89	47.6%
≥39	98	52.4%
RT dose (Gy)		
<57.2	82	43.9%
≥57.2	105	56.1%
SII		
<415.2	96	51.3%
≥415.2	91	48.7%
SIRI		
<0.3	37	19.8%
≥0.3	150	80.2%
NLR		
<1.85	97	51.9%
≥1.85	90	48.1%
PNI		
<49.5	109	58.3%
≥49.5	78	41.7%
PLR		
<231.1	163	87.2%
≥231.1	24	12.8%
PAR		
<8.0	159	85.0%
≥8.0	28	15.0%

SCLC, small cell lung cancer; RE, radiation esophagitis; CCRT, concurrent chemoradiotherapy; RT, radiotherapy; SII, systemic immune-inflammation index; SIRI, system inflammation response index; NLR, neutrophil-lymphocyte ratio; PNI, prognostic-nutrition index; PLR, platelets-lymphocyte radio; PAR, platelets-albumin ratio.

### Univariate and multivariate analyses

The Univariate and multivariate analysis results were shown in **Table 2**. The potential factors for predicting RE were as follows: CCRT (OR, 3.402; p < 0.001; 95% CI, 1.850-6.257), chemotherapy cycle (OR, 0.609; p = 0.097; 95% CI, 0.340-1.093), SIRI (OR, 0.480; p = 0.048; 95% CI, 0.231-0.999), PNI (OR, 0.581; p = 0.073; 95% CI, 0.321-1.052), PLR (OR, 2.794; p = 0.026; 95% CI, 1.131-6.900), PAR (OR, 2.536; P = 0.029; 95% CI, 1.101-5.845) in the univariate analysis. Risk factors with p < 0.10 in univariate analysis were incorporated into the multivariate analysis to determine independent predictors of the occurrence of RE. Multivariate analysis indicated that CCRT (p < 0.001; OR, 3.380; 95% CI, 1.767-6.465], SIRI (p = 0.047; OR, 0.436; 95% CI, 0.192-0.989), and PAR (p = 0.036; OR, 2.907; 95% CI, 1.071-7.891) were independent predictors of grade ≥2 RE. Finally, these independent predictors were utilized to construct the nomogram.

**Table 2:** Univariate and multivariate logistic regression analysis of the clinical, dosimetric factors and inflammation indexes in predicting grade ≥2 RE.

Parameters	Univariate Analysis			Multivariate Analysis		
	OR	95%CI	P	OR	95%CI	P
Gender						
Male vs. Female	0.948	0.306-2.936	0.926			
Age (years)						
<55 vs.≥55	0.641	0.336-1.225	0.178			
Smokers						
Yes vs. No	0.900	0.505-1.601	0.719			
CCRT						
Yes vs. No	3.402	1.850-6.257	<0.001	3.380	1.767-6.465	<0.001
Chemotherapy cycle						
<4 vs. ≥4	0.609	0.340-1.093	0.097	0.943	0.487-1.826	0.861
Stage						
Limited vs Extensive	0.951	0.511-1.769	0.874			
Duration of radiotherapy (day)						
<39 vs. ≥39	0.769	0.432-1.371	0.374			
RT dose (Gy)						
<57.2 vs. ≥57.2	0.635	0.354-1.137	0.127			
SII						
<415.2 vs. ≥415.2	1.430	0.802-2.550	0.226			
SIRI						
<0.3 vs. ≥0.3	0.480	0.231-0.999	0.048	0.436	0.192-0.989	0.047
NLR						
<1.85 vs. ≥1.85	0.743	0.416-1.325	0.314			
PNI						
<49.5 vs. ≥49.5	0.581	0.321-1.052	0.073	0.602	0.303-1.198	0.149
PLR						
<231.1 vs. ≥231.1	2.794	1.131-6.900	0.026	1.783	0.601-5.291	0.298
PAR						
<8.0 vs. ≥8.0	2.536	1.101-5.845	0.029	2.907	1.071-7.891	0.036

RE, radiation esophagitis; OR, odds ratio; 95% confidence interval; CCRT, concurrent chemoradiotherapy; RT, radiotherapy; SII, systemic immune-inflammation index; SIRI, system inflammation response index; NLR, neutrophil-lymphocyte ratio; PNI, prognostic-nutrition index; PLR, platelets-lymphocyte radio; PAR, platelets-albumin ratio.

### Development and validation of a nomogram

**Figure 1:**
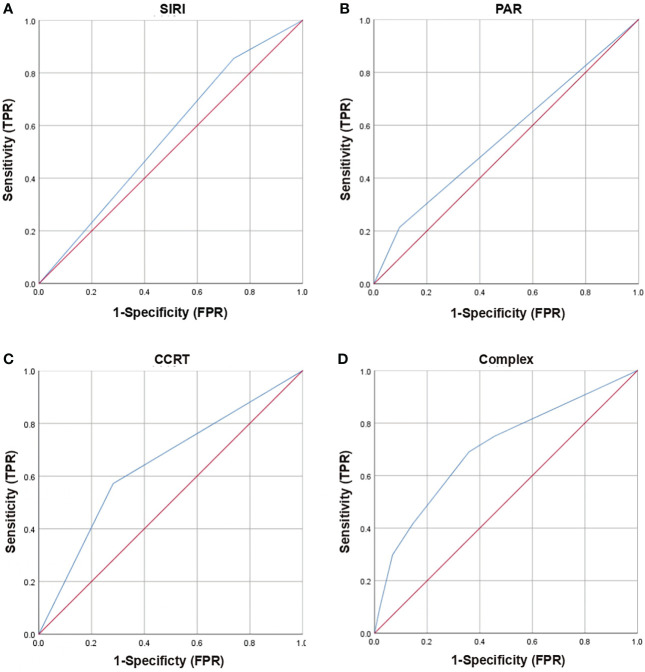
Receiver operating characteristic (ROC) curves of clinical and dosimetric factors (CCRT), inflammation index (SIRI, PAR) and complex (SIRI, PAR, CCRT) for grade ≥2 RE. **(A)** ROC curves of SIRI; **(B)** ROC curves of PAR; **(C)** ROC curves of CCRT; **(D)** ROC curves of Complex (SIRI, PAR, CCRT); RE, radiation esophagitis; SIRI, system inflammation response index; PAR, platelets-albumin ratio; CCRT, concurrent chemoradiotherapy; TPR, true positive rate; FPR, false positive rate.

**Figure 2:**
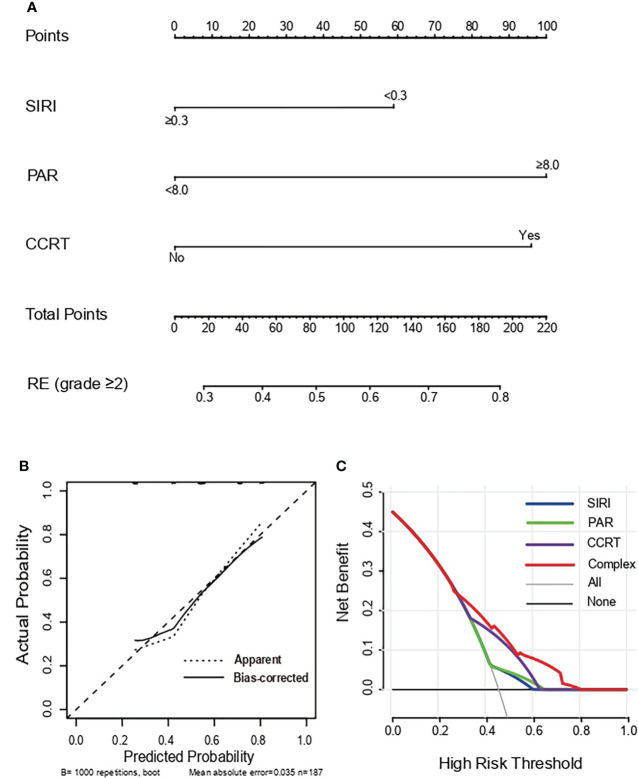
Nomogram, Calibration curve and Decision curve predicting the development of grade ≥2 RE. **(A)** A nomogram incorporated SIRI, PAR and CCRT in SCLC patients. **(B)** Calibration curves of the occurrence of grade ≥2 RE for SCLC patients in the nomogram. The X-axis and Y-axis represent the predicted probability and actual probability respectively. The 45° dotted line showed the best predicted value. **(C)** Decision curves of the occurrence of grade ≥2 RE for SCLC patients in the nomogram. The X-axis and Y-axis represent the threshold probabilities and the net benefit respectively which is computed by adding the true positives and subtracting the false positives. The green, blue, purple and red lines display the net benefit of the SIRI, PAR, CCRT and Complex, respectively. RE, radiation esophagitis; SIRI, system inflammation response index; PAR, platelets-albumin ratio; CCRT, concurrent chemoradiotherapy.

Based on multivariate analysis results, concurrent chemoradiotherapy, SIRI, and PAR were included in the nomogram model. The receiver operating curve (ROC) curves of CCRT, SIRI, PAR, and the complex (CCRT, SIRI, and PAR) were displayed in **Figure 1**. The AUC value of the nomogram was 0.702 (95% CI, 0.626-0.778), which was greater than each factor [CCRT: 0.645, 95% CI, 0.565-0.725; SIRI: 0.558, 95% CI, 0.475-0.642; PAR: 0.559, 95% CI, 0.475-0.642 (**Figure 2A**)]. It is proved that the model had preferable discrimination ability. The calibration curve displayed good consistency between the actual and predicted probability of occurrence of RE (**Figure 2B**). Finally, the DCA demonstrated favorable positive net benefits of the nomogram in the threshold probabilities, indicating a satisfactory clinical benefit of the model (**Figure 2C**).

### Correlation between inflammation indexes

**Figure 3:**
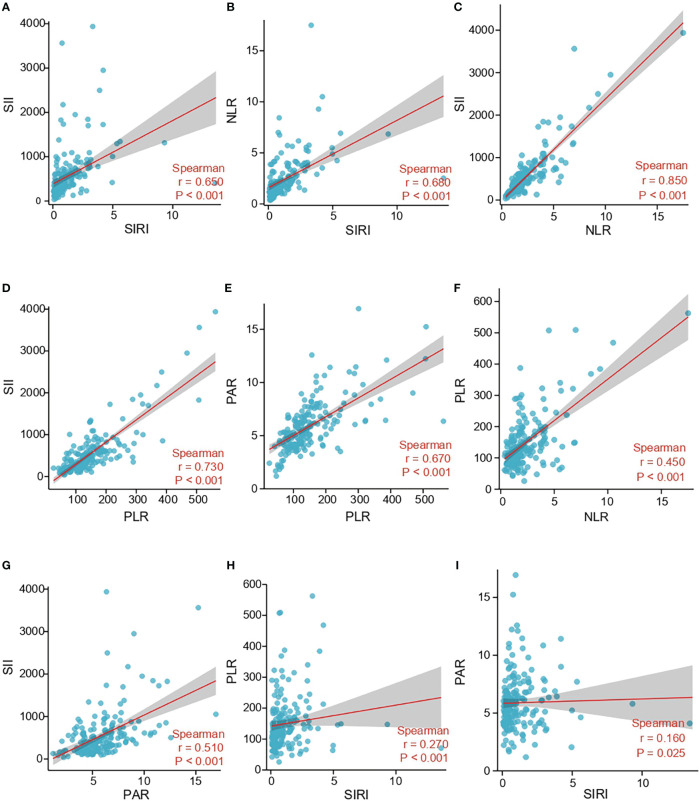
Correlation between SII, SIRI, NLR, PLR and PAR in the study cohort. **(A)** The correlation between SII and SIRI. **(B)** The correlation between NLR and SIRI. **(C)** The correlation between SII and NLR. **(D)** The correlation between SII and PLR. **(E)** The correlation between PAR and PLR. **(F)** The correlation between PLR and NLR. **(G)** The correlation between SII and PAR. **(H)** The correlation between PLR and SIRI. **(I)** The correlation between PAR and SIRI; SII, systemic immune-inflammation index; SIRI, system inflammation response index; NLR, neutrophil-lymphocyte ratio; PLR, platelets-lymphocyte radio; PAR, platelets-albumin ratio.

Spearman correlation was further applied to analyze the correlation among SII, SIRI, NLR, PLR, and PAR. Spearman’s analyses showed that there were strong positive correlations between SIRI and SII (r=0.650, p < 0.001), SIRI and NLR (r=0.680, p < 0.001), NLR and SII (r=0.850, p < 0.001), PLR and SII (r=0.730, p < 0.001), PLR and PAR (r=0.670, p < 0.001) (**Figures 3A-E**). Secondly, spearman’s analyses indicated moderate positive correlations between NLR and PLR (r=0.450, p < 0.001), PAR and SII (r=0.510, p < 0.001) (**Figures 3F-G**). Finally, spearman’s analyses displayed that SIRI was weakly positively correlated with PLR (r=0.270, p<0.001) and PAR (r=0.160, p = 0.025) (**Figures 3H, I**).

## Discussion

Chemoradiotherapy is the primary treatment regimen in patients with small cell lung cancer (SCLC), especially limited-stage small cell lung cancer (LS-SCLC) (13). In extensive-stage small cell lung cancer (ES-SCLC), if patients had a complete or partial response to the initial systemic treatment, thoracic radiotherapy can be performed (14). Surgery is available in only 2-5% of patients, and a study has shown that radiotherapy has better survival outcomes than patients who underwent surgery (15). Therefore, chemoradiotherapy has become the treatment of choice for most patients. Although SCLC is sensitive to radiotherapy and chemotherapy, local recurrence rates and survival prognosis are still unsatisfactory (16). Several clinical studies suggested that shortening overall treatment duration or increasing radiation dose may lead to better survival outcomes (17–19). However, this improved survival rate comes at a cost, namely increased toxicity, especially radiation esophagitis (RE) (17, 20). Consequently, it is essential to recognize some of the factors associated with severe RE.

In this study, we established a nomogram model for grade ≥2 RE in SCLC patients who underwent chemoradiotherapy. We investigated 14 factors associated with the risk of grade ≥2 RE, including gender, age, smokers, CCRT, chemotherapy cycle, stage, duration of radiotherapy, RT dose, SII, SIRI, NLR, PNI, PLR, and PAR. Our data showed that CCRT, SIRI, and PAR were significantly correlated with grade ≥2 RE. As far as we know, the combination of CCRT, SIRI, and PAR for chemoradiotherapy in SCLC patients is the first nomogram model reported to assess the occurrence of RE. The model demonstrated remarkable good consistency and discriminative ability between the predicted risks and observed results. Bootstrap validation proved the stability of the model for similar populations in the future. Decision curve analysis (DCA) also showed potential clinical utility for future clinical practice.

The relationship between concurrent chemoradiotherapy (CCRT) and RE has been documented (11, 21–23). Compared with patients who received sequential chemoradiotherapy or radiotherapy alone, patients who received CCRT had an approximately five-fold increased risk of developing acute RE (11). Similarly, CCRT was significantly associated with grade ≥2 RE in our multivariate analysis. Due to the rapid doubling time and high aggressiveness of SCLC, CCRT had a favorable survival outcome in patients receiving chemoradiotherapy. However, for malignant tumors involving the esophagus in the thoracic radiation field, CCRT increased the incidence and degree of acute esophageal injury (24). One possible reason is that the daily administration of platinum keeps the concentration of platinum in the tissue above the threshold level for radiation enhancement, which leads to a relatively high frequency of RE. And we must keep in mind that RE may damage the patients’ condition later. Esophageal stricture has been reported after a long incubation period (25, 26). It is still an unsolved problem to achieve satisfactory therapeutic effects while reducing toxic side effects. One article reported that conserving the esophageal technique can limit the occurrence of RE in patients with non-small cell lung cancer receiving CCRT without compromising local control (27). Therefore, we hope to develop a more comprehensive individualized treatment plan for chemoradiotherapy patients to reduce or avoid treatment-related toxicity.

A large amount of radiation toxicities has been assumed to be caused by radiation-induced inflammatory responses (28, 29). And previous evidence has shown that some serum inflammatory markers, including interleukin (ILs) and transforming growth factor (TGF-β) were correlated with RE (30–32). These studies emphasize the role of inflammation in the toxicity of RT. Unfortunately, because these indicators were not routinely monitored in the clinic, they were not widely utilized in clinical practice. Whereas, the inflammatory indicators including SII, SIRI, NLR, PLR, and PAR were simply measured by neutrophils, platelets, monocytes, lymphocytes, and albumins, which could be easily and conventionally measured during treatment. At present, the role of inflammatory index and incidence of RE in SCLC undergoing chemoradiotherapy has not been reported. To the best of my knowledge, this is the first study to assess the association between RE and inflammatory index in patients with SCLC. Our results demonstrated that SIRI and PAR were independent predictive factors of the grade ≥2 RE. SIRI < 0.3 and PAR > 8.0 were significantly associated with the occurrence of grade ≥2 RE. Interestingly, the result was consistent with other studies of lung cancer patients treated with chemoradiotherapy. A study has indicated that concurrent chemoradiotherapy and neutropenia were significantly associated with grade ≥2 RE (33). One study suggested that high platelet counts and low hemoglobin levels before radiotherapy were closely related to the occurrence of RE (34). The decrease of neutrophils and platelets could reliably reflect the systemic inflammation of cancer patients (35). Albumin synthesis can be inhibited by reduced protein intake or acute phase reaction, and inflammation was an important factor leading to the decrease of albumin synthesis (36). In this study, we preliminarily demonstrated a close relationship between inflammation index and RE.

It should not be ignored that radiation esophagitis (RE) is a common adverse effect of thoracic radiotherapy, affecting the patient’s therapeutic effect and quality of life. Therefore, it is necessary for SCLC patients receiving radiotherapy to identify this toxicity as early as possible. Although there are a few predictive models based on clinical and dosimetric factors with good discriminative ability, the addition of new biomarkers can improve the predictive power of RE. More importantly, find biomarkers that are readily available and clinically useful. The accurate prediction of RE is critical for facilitating individualized radiation doses and maximizing therapeutic benefits.

A few shortcomings should be pointed out here. First of all, there might be a selection bias in our research due to the retrospective research. Additionally, many patient factors were not included in the study in this heterogeneous population. Some of these features may be associated with the occurrence of RE in individuals. Third, toxicity analysis of different grades of RE was not performed in our study. Finally, the sample size of this study was small, so a large number of queues were required to further construct and verify the nomogram to predict RE.

## Conclusion

To sum up, our research showed that CCRT, SIRI, and PAR were independent predictive factors for grade ≥2 RE in SCLC patients who underwent chemoradiotherapy. We developed and validated a predictive model using these factors. The developed nomogram with superior prediction ability can be used as a quantitative model to predict RE. Any newly developed predictive model will need further validation before it can be advanced to clinical use.

## Data availability statement

The data that support the findings of this study are available from the corresponding author upon reasonable request.

## Author contributions

JL and JQ designed this study. JQ and DK contributed to the data collection. HCL, YY, and HL analyzed the data. JL supervised the study. JQ, YY, QZ, LL, HZ, and HL wrote the manuscript. All authors contributed to the article and approved the submitted version.

## Funding

The project was supported by the National Clinical Key Specialty Construction Program, Fujian provincial Clinical Research Center for Cancer Radiotherapy and Immunotherapy (Grant number 2020Y2012), and Fujian Clinical Research Center for Radiation and Therapy of Digestive, Respiratory and Genitourinary Malignancies (2021Y2014).

## Acknowledgments

We would like to thank all the investigators and patients and acknowledge the work of Jiancheng Li, which remarkably improved the quality of this paper.

## Conflict of interest

The authors declare that the research was conducted in the absence of any commercial or financial relationships that could be construed as a potential conflict of interest.

## Publisher’s note

All claims expressed in this article are solely those of the authors and do not necessarily represent those of their affiliated organizations, or those of the publisher, the editors and the reviewers. Any product that may be evaluated in this article, or claim that may be made by its manufacturer, is not guaranteed or endorsed by the publisher.

## Abbreviations

RE, radiation esophagitis; SCLC, small cell lung cancer; ROC, receiver operating curve; AUC, the area under the receiver operating curve; DCA, decision curve analysis; CCRT, concurrent chemoradiotherapy; SIRI, system inflammation response index; PNI, prognostic-nutrition index; PLR, platelets-lymphocyte radio; PAR, platelets-albumin ratio; OR, odds ratio; CI confidence interval; LS-SCLC, limited-stage small cell lung cancer; ES-SCLC, extensive-stage small cell lung cancer; IMRT, intensity modulated radiation therapy; 3D-CRT 3-dimensional conformal radiation therapy; CT, computed tomography; RT, radiotherapy; GTV, gross tumor volume; CTV, clinical tumor volume; PTV, planned tumor volume; OARs, organs at risk; RTOG, radiation therapy oncology group; ROTRC, European organization for research and treatment of cancer; NCCN, national comprehensive cancer network; SII, systemic immune-inflammation index; NLR, neutrophil-lymphocyte ratio; ILs, interleukin; TGF-β, transforming growth factor.
